# Longitudinal Association between Periodontitis and the Risk of Hypertension

**DOI:** 10.1155/2023/2644623

**Published:** 2023-06-17

**Authors:** John B. Aremu, Cynthia M. Pérez, Kaumudi J. Joshipura

**Affiliations:** ^1^College of Nursing and Health Professions, Arkansas State University, Jonesboro, AR, USA; ^2^Department of Biostatistics & Epidemiology, Rutgers School of Public Health, Piscataway, NJ, USA; ^3^Department of General Dentistry, Boston University Henry M. Goldman School of Dental Medicine, Boston, MA, USA; ^4^Department of Biostatistics and Epidemiology, Graduate School of Public Health, University of Puerto Rico Medical Sciences Campus, San Juan, PR, USA; ^5^Department of Epidemiology, Harvard T. H. Chan School of Public Health, Boston, MA, USA; ^6^School of Public Health, Ahmedabad University, Gujarat, India

## Abstract

**Objectives:**

Hypertension poses a major public health challenge due to its association with increased risk of heart disease, chronic kidney disease, and death. The objective of this study is to evaluate the longitudinal association between periodontitis and the risk of hypertension.

**Methods:**

Using a cohort study design, 540 participants free of diagnosed hypertension/prehypertension in the San Juan Overweight Adults Longitudinal Study and with complete 3-year follow-up data were included. Periodontitis was classified according to the 2012 Centers for Disease Control and Prevention/American Academy of Periodontology definition. Participants were considered to have developed hypertension if they reported physician-diagnosed hypertension over the follow-up period or had average systolic blood pressure (SBP) ≥140 mm Hg or diastolic blood pressure (DBP) ≥90 mm Hg at follow-up. Participants free of diagnosed hypertension or prehypertension and with normal BP at baseline (SBP < 120 mm Hg and DBP < 80 mm Hg) were considered to develop prehypertension if they had SBP between 120 and 139 mm Hg or DBP between 80 and 89 mm Hg at follow-up. An additional (secondary) outcome was defined as the development of prehypertension/hypertension over the follow-up period among participants who had normal BP at baseline. We used Poisson regression, adjusting for age, sex, smoking status, physical activity, alcohol intake, diabetes, waist circumference, and family history of hypertension.

**Results:**

One hundred and six (19.6%) participants developed hypertension, and 58 of the 221 with normal BP (26%) developed prehypertension/hypertension. There was no consistent association between periodontitis and the risk of developing hypertension. However, people with severe periodontitis had an increased incidence of prehypertension/hypertension (multivariate incidence rate ratios: 1.47; 95% confidence interval: 1.01, 2.17) than people without periodontitis after adjusting for confounders.

**Conclusion:**

There was no association between periodontitis and hypertension in this cohort study. However, severe periodontitis was associated with an increased risk of prehypertension/hypertension.

## 1. Introduction

Hypertension pose a significant public health problem due to its association with heart disease, chronic kidney disease, stroke, and death [1–4]. Periodontitiss is a chronic inflammatory condition that affects the supporting structures of the teeth (gums, alveolar bone, cementum, and periodontal ligament). It is characterized by soft and hard tissue loss, including clinical attachment loss (CAL), bone loss, gingival recession, and pocket formation. The prevalence of periodontitis in the adult population is over 45%, affecting almost 65 million adults 30 years and older [5–7].

The literature reports associations between chronic periodontitis and cardiovascular diseases (CVDs), including hypertension [8–12]. A consensus report on periodontitis and CVD also highlights the mechanistic associations between periodontitis and CVD. Few studies reported an association between periodontitis and hypertension [9, 10, 12, 13], while some reported no association [14–17]. Most of these publications are cross-sectional [9, 12, 14, 18–21], a few are prospective [13, 15–17], and the studies use varying definitions of periodontitis and hypertension, making it difficult to compare results across studies.

Recent reviews of epidemiological studies assessing the relationship between hypertension and periodontitis reported inconsistent findings. Two systematic reviews suggested a positive association between periodontitis and hypertension [11, 22]. However, they were inconclusive about the biological mechanisms and causal relationship, and the inadequate number of prospective studies was a major limitation. Periodontitis may lead to increased chronic systemic inflammation and endothelial dysfunction, which in turn may lead to an increased risk of hypertension [8, 10, 20]. Nevertheless, these associations may also be explained by shared risk factors. Hypertension and periodontitis share several common risk factors, including obesity, increasing age, dyslipidemia, and inflammatory biomarkers such as tumor necrosis factor-alpha, cytokines, and C-reactive protein [19].

A cohort study evaluating the relationship between hypertension and periodontal disease among men showed no significant association between incident hypertension and periodontal disease at baseline and during 20 years of follow-up (relative risk (RR): 1.04; 95% confidence interval (CI): 0.98, 1.10) [16]. Although this study was large and adequately powered, the periodontal disease was self-reported. Another large, short-term cohort study found a significant association between periodontal disease and incidence of hypertension [9] but did not include validated clinical measures for assessing periodontitis and hypertension. It also lacked generalizability since it only included young university students who are less likely to have periodontitis.

The intricate relationship between periodontitis and hypertension has mostly been investigated by cross-sectional studies, majority of which were not adequately powered and did not utilize standard clinical measures of assessing of periodontitis and/or hypertension. In this study, we evaluated the longitudinal association between periodontitis and the risk of hypertension within a large cohort using standard assessments of periodontitis and hypertension.

## 2. Methods

### 2.1. Study Design and Population

The study was conducted using the San Juan Overweight Adults Longitudinal Study (SOALS), the design and methods have been described in detail in previous publications [8, 18, 23–25]. SOALS recruited overweight and obese adults aged 40–65 free of physician diagnosed hypertension at baseline. Exclusion criteria were (i) pregnancy, (ii) use of orthodontic appliances, (iii) bleeding disorders, (iv) history of CVD or other major health conditions, (v) severe health conditions/disabilities, (vi) anticoagulation therapy, (vii) needing antibiotic prophylaxis before dental procedures, (viii) having cardiac pacemaker or prosthetic hip/joint replacement, (ix) having less than four teeth, (x) inability to participate in the study, and (xi) plans to relocate from San Juan within 3 years. The University of Puerto Rico Medical Sciences Campus Institutional Review Board approved this study (Protocol Number: A4840310), and participants gave informed consent. This study follows the STROBE guidelines [26–28].

**Figure 1:**
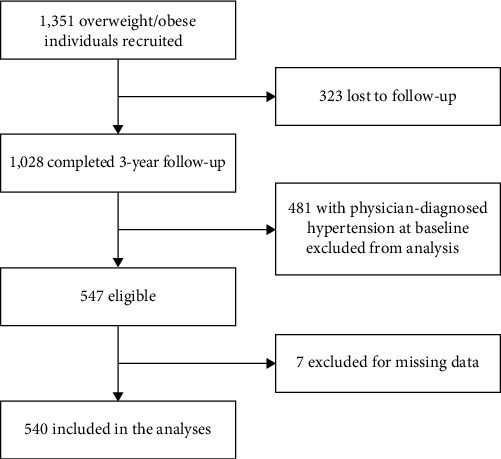
Flowchart of the study population.

Recruitment of participants and data collection started in 2011 [18, 23, 29]; 1,351 completed the baseline exams. Participants were followed for 3 years until 2016, during which 1,028 (76%) completed the follow-up exam (Figure 1). We excluded from the analyses 481 people with physician-diagnosed hypertension and those on blood pressure (BP) medication at baseline. Out of the 547 eligible participants, seven people with missing data on key covariates were excluded. The remaining 540 participants were included in the analyses.

### 2.2. Outcomes

Self-reported physician-diagnosed hypertension was ascertained through interviewer-administered questionnaires at baseline and at 3-year follow-up. BP was assessed using the gold standard Korotkoff auscultatory technique with a mercury sphygmomanometer and automated BP machines at baseline and at follow-up [23]. Measurements from mercury sphygmomanometer were used in the analyses. BP was measured after 5 min of rest. Measurements were repeated three times with 1-min intervals and averaged to obtain each participant's mean systolic blood pressure (SBP) and diastolic blood pressure (DBP).

Participants without hypertension at baseline were considered to develop hypertension if they reported physician-diagnosed hypertension over the follow-up period or had average SBP ≥ 140 mm Hg or DBP ≥ 90 mm Hg at follow-up. Since the study was conducted before the new ACC/AHA high BP guidelines in 2017 [23], we assumed that physicians used the prior classification when the diagnosis was performed. Participants free of diagnosed hypertension/prehypertension and normal BP at baseline (SBP < 120 mm Hg and DBP < 80 mm Hg) were considered to develop prehypertension if they had SBP between 120 and 139 mm Hg or DBP between 80 and 89 mm Hg at follow-up.

### 2.3. Exposure: Periodontitis

A full-mouth periodontal examination was performed at baseline and 3-year follow-up using the NHANES procedures [29–31]. Dentists were trained and calibrated to conduct an oral examination to assess periodontal pocket, CAL, dental caries, plaque score, and bleeding on probing (BOP). This training was provided by Dr. Bruce Dye, an NHANES reference examiner [18]. Plaque score was evaluated on four surfaces on six teeth and averaged. Periodontitis may progress to tooth loss, so we assessed the baseline number of teeth by comparing those with at least 25 teeth [6, 24, 26–28, 30–32] with those with fewer teeth [1–5, 8–13, 15–17, 22, 23, 29, 33, 34].

Probing pocket depth (PD) and gingival recession were measured at six sites (distobuccal, mid-buccal, mesiobuccal, distolingual, mid-lingual, and mesiolingual) on each of the 28 permanent teeth (excluding third molars) [18]. CAL was calculated by adding PD and gingival recession. Periodontitis was classified according to the 2012 Centers for Disease Control and Prevention/American Academy of Periodontology (CDC/AAP) definition [18]. Severe periodontitis was defined as having two or more interproximal sites with CAL ≥ 6 mm and at least one interproximal site with PD ≥ 5 mm. Moderate periodontitis was defined as having two or more interproximal sites with CAL ≥ 4 mm or two or more interproximal sites with PD ≥ 5 mm. Mild periodontitis was defined as having at least two interproximal sites with CAL ≥ 3 mm, at least two interproximal sites with CAL ≥ 3 mm, and at least two interproximal sites with PD ≥ 5 mm.

To evaluate reliability, examination was conducted by two out of three examiners [31], with an intraclass correlation coefficient of 0.88 (95% CI: 0.83, 0.95) for mean CAL. Similarly, mean PD had an intraclass correlation coefficient of 0.94 (95% CI: 0.90, 0.97). This shows excellent interexaminer reliability.

### 2.4. Covariates

Information on age, sex, family history of hypertension, smoking status, diabetes, physical activity, education, alcohol intake, diet, and salt intake were collected using an interviewer-administered questionnaire. Anthropometric measurements were conducted using the NHANES III procedures [23, 30, 31]. Duplicate measures were taken and averaged.

### 2.5. Statistical Analysis

Descriptive statistics (using mean and SD and frequency/proportions) were computed overall and by periodontitis levels. The development of prehypertension/hypertension during the follow-up period was modeled as the outcome, while periodontitis at baseline was modeled as the exposure. The data follows Poisson's distribution. The association between periodontitis at baseline and risk of prehypertension/hypertension over a 3-year follow-up was evaluated using Poisson regression. Incidence rate ratios (IRR) and 95% CIs were computed with robust standard errors.

Confounding variables were selected at baseline using a priori knowledge of risk factors for hypertension from the literature, including age, sex, smoking status, physical activity, waist circumference, alcohol intake, diabetes, and family history of hypertension. Additional confounders were assessed by utilizing the process of change in estimate. The variables in the base model were used to assess all the other associations. Subgroup analyses were performed to evaluate whether the association between periodontitis and pre-hypertension/hypertension varied by age, sex, smoking status, physical activity, BMI, antihypertensive medication use, and alcohol consumption. Missing data were handled using the complete case method.

## 3. Results

Table 1 compares the characteristics of participants who completed the baseline examination with participants retained in the analyses. No major differences between the two groups other than the retention of fewer males, suggesting minimal bias from the exclusions. Among those retained in the analyses, the mean age was 49 years (SD = 6.5), 16.8% were current smokers, 62.5% were physically active, 6.2% had diabetes, 62.7% had a family history of hypertension, and 65.9% had moderate/severe periodontitis. Over the follow-up period, 106 (19.6%) developed hypertension, and 16.1% had prehypertension (data not shown).  Table 2 shows baseline characteristics by periodontitis severity. Proportions of males, smokers, and participants with prediabetes/diabetes were higher among those with severe periodontitis.

In Poisson regression models relating periodontitis with the development of hypertension, baseline periodontitis was not statistically significantly associated with the risk of incident hypertension (Table 3). However, severe periodontitis significantly increased the risk of incident prehypertension/hypertension by 47% (95% CI: 1.01–2.17) after adjusting for age, sex, smoking status, family history of hypertension, diabetes, waist circumference, alcohol consumption, and physical activity). We explored whether selected baseline covariates modified the association between periodontitis and hypertension (Table 4) and prehypertension/hypertension (Table 5). Participants 55 years and over with severe periodontitis had a four-fold elevated risk of incident hypertension than those below 55 years without periodontitis (IRR: 4.46, 95% CI: 1.40–14.22) after adjusting for age, sex, smoking status, physical activity, alcohol consumption, waist circumference, diabetes, and family history of hypertension. Similarly, participants 55 years and over with severe periodontitis had about a three-fold increased risk of developing incident prehypertension/hypertension than those aged 55 or younger without periodontitis (IRR: 2.98, 95% CI: 1.19, 7.49). In addition, females with severe periodontitis had a higher risk of incident prehypertension/hypertension (IRR: 1.55, 95% CI: 1.03–2.34) than males without periodontitis after adjusting for age, smoking status, physical activity, alcohol consumption, waist circumference, diabetes, and BP medication. Obese individuals with severe periodontitis also had a higher risk of incident prehypertension/hypertension (IRR: 1.55, 95% CI: 1.02–2.35) than normal weight individuals without periodontitis after adjusting for age, sex, smoking status, physical activity, alcohol consumption, waist circumference, diabetes, and BP medication.

Additional adjustments were also conducted for other variables like visits to the dentist (including the reason for the visit), periodontal therapy during the follow-up period, brushing frequency, use of mouthwash, flossing, and income level. These adjustments did not result in a significant change in the IRR (change was below 10% for every association).

## 4. Discussion

There was no association between periodontitis and risk of incident hypertension over the 3-year follow-up period. After adjusting for major confounders, severe periodontitis was associated with a statistically significant increased risk of incident prehypertension/hypertension. Participants included in the final analysis were similar to those who completed the baseline exam with regard to key risk factors for hypertension, suggesting minimal selection bias from loss to follow-up. However, obesity, prediabetes, and diabetes were lower at follow-up than baseline. This may likely be due to the study impacting healthy behaviors rather than differential loss to follow-up and is unlikely to bias our findings.

We found a four-fold increase in the risk of hypertension among participants with severe periodontitis who were at least 55 years compared to those without periodontitis below 55 years. The higher risk observed among older individuals with severe periodontitis may be because the extent of periodontal destruction is directly related to age, and older people may experience delayed healing of periodontal tissues [35]. Older individuals also tend to have an increased presence of inflammatory biomarkers, which may increase their risk of developing hypertension [35]. Similarly, those aged 55 years and over and obese people with severe periodontitis had an increased risk of developing combined prehypertension/hypertension, which may be due to the prolonged inflammation among older and obese individuals.

Previous studies used the self-reported incidence of periodontal disease, Russell's Periodontal Index, or Community Periodontal Index to assess periodontitis. Several classifications of periodontal diseases exist in the literature, including the 1999 classification [36, 37], updated in 2015 by the AAP and modified again in 2017/2018 at the *World Workshop on the Classification of Periodontal and Peri-implant Diseases and Conditions* [38]. For population-based studies, periodontitis is defined in multiple ways with no consensus on the best measures. Our study used the CDC/AAP case definition, which, although designed for surveillance studies, is often used in epidemiological studies.

The relationship between periodontal disease and the risk of developing hypertension was evaluated among 31,543 participants of the Health Professionals' Follow-up Study (HPFS) over a 20-year follow-up period [16]. Periodontal disease was self-reported by the participants, and hypertension was assessed using self-reported physician-diagnosed hypertension as the outcome, which was validated in that population [23]. No significant associations were observed between periodontal disease at baseline and incident hypertension during follow-up (RR: 1.01; 95% CI: 0.96–1.05). Our findings are similar to the cohort study conducted among participants of the HPFS, even though it was conducted among males only, and the follow-up period was more extended than ours.

Another cohort study comprising 2,588 university students in Japan aged 18–27 years followed for 3 years revealed a significant association between periodontal disease and hypertension, defined as SBP ≥ 140 mm Hg or DBP ≥ 90 mm Hg (odds ratio (OR): 2.74; 95% CI: 0.51–1.70), but there was no association between the presence of periodontal disease (defined as the presence of BOP ≥ 30% at baseline and probing PD ≥ 4 mm) and development of prehypertension (OR: 0.93; 95% CI: 0.51–1.70) [9]. In another study of older adults aged 70–97 years [21], severe periodontitis was found to be associated with high BP (OR: 2.35; 95% CI: 1.08–5.14), but the study only controlled for age and sex.

The findings of our study contradict those of Kawabata et al. [9] showing an association between periodontal disease and hypertension. This is not surprising as their study was restricted to young university students aged 18–27 years, and periodontitis was defined as PD ≥ 4 mm.

Even though periodontitis was not associated with an elevated risk of developing hypertension, we found that severe periodontitis was associated with an increased risk of prehypertension/hypertension. The reason for this finding is unknown; however, a potential explanation could be an increase in sample size when we combined those with prehypertension and hypertension, increasing the statistical power to detect such an association. Out of the 114 participants with severe periodontitis, 16 had hypertension, while 41 developed prehypertension/hypertension at the end of the follow-up period (data not shown).

This is one of the first studies to assess the longitudinal associations between standard measures of periodontitis and the risk of prehypertension/hypertension. Another strength of this study is the standardized classification of periodontitis, which increases the internal validity. Significant limitations of our study include a relatively short follow-up period of 3 years. Additionally, despite having a large sample size and almost 80% retention rate, only a few participants developed hypertension, limiting the statistical power of the study. Furthermore, the findings are not generalizable to the whole US population because it was conducted among overweight/obese Hispanic individuals aged 40–65 and free of major chronic diseases.

Despite these limitations, our findings suggest a potential association between periodontitis and an increased risk of developing prehypertension/hypertension. Even though our study suggests no association between periodontitis and the risk of hypertension, it highlights the need for conducting larger, well-designed longitudinal studies with more extended follow-up periods to corroborate our findings and to evaluate potential mediators of the association. Our study findings are an integral step toward recognizing the complex interaction between periodontal diseases and cardiovascular conditions and suggest that improving dental health may be a plausible way of reducing the risk of increased BP.

## Figures and Tables

**Table 1:** Descriptive statistics of baseline characteristics of study participants (% or mean ± SD).

	All baseline (*n* = 1,028)	Retained in analysis (*n* = 540)
Age (years), mean ± SD	50.7 ± 6.8	49.0 ± 6.5
Male	27.0	22.0
Smoking	16.5	16.8
Former	18.1	20.9
Current	16.5	16.8
Annual income (<$20,000)	54.3	56.5
Education		
<High school	11.2	10.8
High school	44.0	42.4
≥Some college	44.8	46.0
Physical activity		
No physical activity	36.4	37.5
Some physical activity	7.3	6.8
Vigorous physical activity	56.3	55.7
Obese	64.1	57.4
Alcohol consumption (g/day)	2.3 ± 5.8	2.2 ± 6.1
Waist circumference (cm)	106.3 ± 14.1	104.1 ± 13.0
Prediabetes	52.5	47.9
Diabetes	7.5	6.2
Family history of hypertension	63.9	62.7
Plaque score	0.8 ± 0.6	0.8 ± 0.6
Number of sites with bleeding on probing	12.4 ± 12.0	12.7 ± 12.2
Always add salt to food	4.8	4.8
Elevated blood pressure	31.3	31.8
Periodontitis (moderate/severe)	67.2	65.9
Mean PD (mm)	1.4 ± 0.9	1.5 ± 1.0
Mean CAL (mm)	1.8 ± 1.2	1.7 ± 1.2
Dental caries	9.2 ± 0.8	8.9 ± 1.2
Number of teeth	23.5 ± 4.4	23.8 ± 4.2

**Table 2:** Baseline characteristics by periodontitis severity.

Sociodemographic and clinical characteristics	Level	Mild periodontitis	Moderate periodontitis	Severe periodontitis
Number of participants		183	243	114

Age (years), mean ± SD		47.0 (5.6)	50.0 (6.8)	50.1 (6.6)

Male		16.9%	21.8%	30.7%

Smoking	Current	13.1%	21.8%	31.6%
	Former	16.4%	16.9%	21.1%
	None	70.5%	61.3%	47.4%

Annual income	<$20,000	61.7%	58.0%	53.2%
	>$20,000	38.3%	42.0%	46.8%

Education	<High school	11.4%	10.6%	12.1%
	High school	42.7%	49.0%	44.9%
	≥Some college	45.9%	40.4%	43.0%

Physical activity	None	36.6%	35.8%	41.2%
	Some	6.0%	6.6%	8.8%
	Vigorous	57.4%	57.6%	50.0%

BMI (SD)		32.4 (5.4)	32.5 (6.1)	32.4 (5.5)

Alcohol consumption (g/day), mean (SD)		1.9 (7.1)	2.1 (5.6)	2.7 (5.2)

Waist circumference, mean (SD)		103.3 (12.8)	103.9 (12.8)	105.0 (13.0)

Diabetes	Normoglycemic	54.1%	44.0%	36.8%
	Prediabetes	42.1%	48.6%	55.3%
	Diabetes	3.8%	7.4%	7.9%

Family history of hypertension	No	36.1%	44.9%	43.9%
	Yes	63.9%	55.1%	56.1%

Plaque score		0.7 (0.5)	0.8 (0.3)	0.9 (0.6)

BOP, mean (SD)		0.2 (0.2)	0.3 (0.2)	0.5 (0.3)

Always add salt to food	Yes	4.7%	4.8%	4.8%
	No	95.3%	95.2%	95.2%

Elevated blood pressure	Yes	32.6%	35.8%	36.0%
	No	69.4%	64.2%	64.0%

PD, mean (SD)		1.6 (0.3)	2.0 (0.4)	3.0 (1.0)

CAL (mm), mean (SD)		1.0 (0.3)	1.9 (0.7)	3.5 (1.6)

Dental caries, mean (SD)		10.2 (9.1)	9.8 (8.5)	7.6 (4.6)

Baseline number of teeth	1–10	1.1%	1.6%	1.8%
	11–16	2.7%	4.5%	6.1%
	17–24	33.9%	44.0%	51.8%
	25–32	62.3%	49.8%	40.4%

**Table 3:** Multivariable-adjusted associations (incidence rate ratios) relating periodontitis with the development of hypertension and combined prehypertension/hypertension. ^*∗*^

	Hypertension			Prehypertension/hypertension		
	IRR	95 (%) CI	*P*	IRR	95 (%) CI	*P*
Periodontitis^+^						
None	1.00	–	–	1.00	–	–
Mild	1.68	0.87–3.25	0.12	1.13	0.68–1.87	0.22
Moderate	0.68	0.39–1.20	0.19	0.94	0.65–1.34	0.10
Severe	1.02	0.54–1.95	0.95	**1.47**	**1.01–2.17**	**0.04**
Mean PD	1.03	0.79–1.35	0.82	1.19	0.90–1.42	0.09
Mean CAL	0.94	0.79–1.50	0.51	0.99	0.87–1.36	0.24
Number of teeth at baseline						
25–32	1.00	–	–	1.00	–	–
17–24	0.82	0.54–1.25	0.35	0.89	0.71–1.22	0.29
11–16	0.95	0.37–2.49	0.92	0.94	0.87–1.40	0.78
1–10	1.08	0.26–4.57	0.91	1.11	0.99–2.68	0.87

^*∗*^Adjusted for age, sex, smoking status, family history of hypertension, diabetes, waist circumference, alcohol consumption, and physical activity. ^+^Severe periodontitis was defined as having two or more interproximal sites with CAL ≥ 6 mm and at least one interproximal site with PD ≥ 5 mm. Moderate periodontitis was defined as having two or more interproximal sites with CAL ≥ 4 mm or two or more interproximal sites with PD ≥ 5 mm. Mild periodontitis was defined as having at least two interproximal sites with CAL ≥ 3 mm, at least two interproximal sites with CAL ≥ 3 mm, and at least two interproximal sites with PD ≥ 5 mm. Bold values denotes statistically significant at *P* < 0.05.

**Table 4:** Incidence rate ratios relating periodontitis and incidence of hypertension during the follow-up period within subgroups defined by baseline characteristics.

Subgroups	*N*	IRR^+^ (95% CI)		
		Mild periodontitis	Moderate periodontitis	Severe periodontitis
Age				
<55	424	1.41 (0.70, 2.86)	0.56 (0.29, 1.08)	0.71 (0.32, 1.59)
≥55	116	3.77 (0.32, 4.51)	1.58 (0.57, 4.38)	4.46^*∗*^ (1.40, 14.22)
Sex				
Male	119	2.57 (0.50, 13.29)	0.36 (0.10, 1.37)	0.53 (0.07, 4.28)
Female	421	1.12 (0.49, 2.57)	0.67 (0.37, 1.22)	1.07 (0.53, 2.18)
Smoking				
Never	332	0.86 (0.28, 2.71)	0.46 (0.20, 1.03)	1.10 (0.48, 2.52)
Ever	208	2.17 (0.75, 6.27)	0.91 (0.33, 2.47)	0.79 (0.27, 2.26)
Physical activity				
No	245	0.99 (0.29, 3.60)	0.77 (0.34, 1.76)	1.05 (0.37, 3.03)
Yes	295	2.57^*∗*^ (1.20, 5.50)	0.56 (0.23, 1.41)	0.92 (0.35, 2.40)
BMI				
Overweight	232	1.61 (0.53, 4.88)	0.49 (0.18, 1.34)	0.50 (0.10, 2.43)
Obese	308	2.43^*∗*^ (1.08, 5.50)	0.92 (0.47, 1.79)	1.65 (0.78, 3.51)
Antihypertensive medication use				
No	521	1.59 (0.72, 3.48)	0.70 (0.36, 1.38)	0.88 (0.41, 1.86)
Yes	19	0.70 (0.43, 1.14)	0.69 (0.42, 1.14)	1.53 (0.73, 3.21)
Alcohol consumption				
Not current	297	2.37 (0.99, 5.63)	0.85 (0.41, 1.78)	0.70 (0.20, 2.51)
Current	243	1.26 (0.45, 3.38)	0.46 (0.20, 1.08)	1.08 (0.47, 2.49)

^+^Adjusted for age, sex, smoking status, physical activity, alcohol consumption, waist circumference, diabetes, and family history of hypertension.  ^*∗*^Statistically significant at 0.05 level.

**Table 5:** Incidence rate ratios relating periodontitis and the development of prehypertension/hypertension during the follow-up within subgroups defined by baseline characteristics.

Subgroups	*N*	IRR^+^ (95% CI)		
		Mild periodontitis	Moderate periodontitis	Severe periodontitis
Age				
<55	424	1.11 (0.67, 1.83)	0.93 (0.62, 1.39)	1.27 (0.81, 1.99)
≥55	116	1.22 (0.16, 9.58)	1.16 (0.51, 2.67)	2.98^*∗*^ (1.19, 7.49)
Sex				
Male	119	1.68 (0.62, 4.56)	0.59 (0.22, 1.56)	0.60 (0.18, 2.04)
Female	421	0.84 (0.46, 1.53)	0.92 (0.63, 1.36)	1.55^*∗*^ (1.03, 2.34)
Smoking				
Never	332	0.80 (0.39, 1.63)	0.70 (0.44, 1.10)	1.47 (0.92, 2.36)
Ever	208	2.05 (0.85, 4.96)	1.63 (0.73, 3.63)	1.74 (0.79, 3.84)
Physical activity				
No	245	1.04 (0.43, 2.49)	0.96 (0.54, 1.73)	1.40 (0.71, 2.77)
Yes	295	1.26 (0.69, 2.31)	0.97 (0.59, 1.60)	1.49 (0.91, 2.43)
BMI				
Overweight	232	1.52 (0.63, 3.63)	1.14 (0.59, 2.23)	1.47 (0.67, 3.21)
Obese	308	1.15 (0.62, 2.14)	0.87 (0.57, 1.34)	1.55^*∗*^ (1.02, 2.35)
Antihypertensive medication use				
No	521	1.13 (0.65, 1.98)	0.98 (0.65, 1.47)	1.43 (0.93, 2.21)
Yes	19	0.70 (0.43, 1.14)	0.69 (0.42, 1.14)	1.53 (0.73, 3.21)
Alcohol consumption				
Not current	297	1.36 (0.71, 2.68)	1.11 (0.68, 1.80)	1.11 (0.59, 2.10)
Current	243	1.06 (0.51, 2.23)	0.74 (0.42, 1.30)	1.54 (0.88, 2.71)

^+^Adjusted for age, sex, smoking status, physical activity, alcohol consumption, waist circumference, diabetes, BP medication.  ^*∗*^Statistically significant at 0.05 level.

## Data Availability

Data are available from thecorresponding author upon reasonable request.

## References

[1] OʼBrien E., Pickering T., Asmar R. (2002). Working Group on Blood Pressure Monitoring of the European Society of Hypertension International Protocol for validation of blood pressure measuring devices in adults. *Blood Pressure Monitoring*.

[2] Kearney P. M., Whelton M., Reynolds K., Muntner P., Whelton P. K., He J. (2005). Global burden of hypertension: analysis of worldwide data. *The Lancet*.

[3] Khera R., Lu Y., Lu J. (2018). Impact of 2017 ACC/AHA guidelines on prevalence of hypertension and eligibility for antihypertensive treatment in United States and China: nationally representative cross sectional study. *BMJ*.

[4] Darnaud C., Thomas F., Pannier B., Danchin N., Bouchard P. (2015). Oral health and blood pressure: the IPC Cohort. *American Journal of Hypertension*.

[5] Sanz M., Ceriello A., Buysschaert M. (2018). Scientific evidence on the links between periodontal diseases and diabetes: consensus report and guidelines of the joint workshop on periodontal diseases and diabetes by the International Diabetes Federation and the European Federation of Periodontology. *Diabetes Research and Clinical Practice*.

[6] Armitage G. C. (1999). Development of a classification system for periodontal diseases and conditions. *Annals of Periodontology*.

[7] Update to 1999 Disease Classification (2015). American Academy of Periodontology Task Force Report on the update to the 1999 classification of periodontal diseases and conditions. *Journal of Periodontology*.

[8] Aarabi G., Eberhard J., Reissmann D. R., Heydecke G., Seedorf U. (2015). Interaction between periodontal disease and atherosclerotic vascular disease—fact or fiction?. *Atherosclerosis*.

[9] Kawabata Y., Ekuni D., Miyai H. (2016). Relationship between prehypertension/hypertension and periodontal disease: a prospective cohort study. *American Journal of Hypertension*.

[10] Southerland J. H. (2013). Periodontitis may contribute to poor control of hypertension in older adults. *Journal of Evidence Based Dental Practice*.

[11] Martin-Cabezas R., Seelam N., Petit C. (2016). Association between periodontitis and arterial hypertension: a systematic review and meta-analysis. *American Heart Journal*.

[12] Ahn Y.-B., Shin M.-S., Byun J.-S., Kim H.-D. (2015). The association of hypertension with periodontitis is highlighted in female adults: results from the fourth Korea National Health and Nutrition Examination Survey. *Journal of Clinical Periodontology*.

[13] Rivas-Tumanyan S., Spiegelman D., Curhan G. C., Forman J. P., Joshipura K. J. (2012). Periodontal disease and incidence of hypertension in the health professionals follow-up study. *American Journal of Hypertension*.

[14] Pérez C. M., Muñoz F., Andriankaja O. M. (2017). Cross-sectional associations of impaired glucose metabolism measures with bleeding on probing and periodontitis. *Journal of Clinical Periodontology*.

[15] Bokhari S. A. H., Khan A. A., Leung W. K., Wajid G. (2015). Association of periodontal and cardiovascular diseases: South-Asian studies 2001–2012. *Journal of Indian Society of Periodontology*.

[16] Aguilera E. M., Suvan J., Buti J. (2020). Periodontitis is associated with hypertension: a systematic review and meta-analysis. *Cardiovascular Research*.

[17] Górski B., Nargiełło E., Ganowicz E., Opolski G., Górska R. (2016). The association between dental status and risk of acute myocardial infarction among poles: case–control study. *Advances in Clinical and Experimental Medicine*.

[18] Joshipura K. J., Muñoz-Torres F. J., Dye B. A., Leroux B. G., Ramírez-Vick M., Pérez C. M. (2018). Longitudinal association between periodontitis and development of diabetes. *Diabetes Research and Clinical Practice*.

[19] Belinga L. E. E., Ngan W. B., Lemougoum D. (2018). Association between periodontal diseases and cardiovascular diseases in Cameroon. *Journal of Public Health in Africa*.

[20] Jiménez-Beato G., Machuca-Portillo G. (2005). Heart and periodontal diseases: does evidence exist of association?. *Medicina Oral, Patologia Oral Y Cirugia Bucal*.

[21] Tsioufis C., Kasiakogias A., Thomopoulos C., Stefanadis C. (2011). Periodontitis and blood pressure: the concept of dental hypertension. *Atherosclerosis*.

[22] Türkoğlu O., Barış N., Tervahartiala T., Şenarslan O., Sorsa T., Atilla G. (2014). Evaluation of systemic levels of neutrophilic enzymes in patients with hypertension and chronic periodontitis. *Journal of Periodontology*.

[23] Joshipura K., Muñoz-Torres F., Vergara J., Palacios C., Pérez C. M. (2016). Neck circumference may be a better alternative to standard anthropometric measures. *Journal of Diabetes Research*.

[24] Vandenbroucke J. P., von Elm E., Altman D. G. (2014). Strengthening the reporting of observational studies in epidemiology (STROBE): explanation and elaboration. *International Journal of Surgery*.

[25] Aremu J. B., Pérez C. M., Joshipura K. (2022). *Longitudinal Association between Periodontitis and the Risk of Hypertension*.

[26] Eke P. I., Thornton-Evans G. O., Wei L., Borgnakke W. S., Dye B. A., Genco R. J. (2018). Periodontitis in US adults: national health and nutrition examination survey 2009–2014. *The Journal of the American Dental Association*.

[27] Eke P. I., Dye B. A., Wei L. (2015). Update on prevalence of periodontitis in adults in the United States: NHANES, 2009 to 2012. *Journal of Periodontology*.

[28] Consensus Report: Chronic Periodontitis (1999). 1999 international workshop for a classification of periodontal diseases and conditions. *Annals of Periodontology*.

[29] Joshipura K. J., Muñoz-Torres F. J., Campos M., Rivera-Díaz A. D., Zevallos J. C. (2018). Association between within-visit systolic blood pressure variability and development of pre-diabetes and diabetes among overweight/obese individuals. *Journal of Human Hypertension*.

[30] Andriankaja O. M., Jiménez J. J., Muñoz-Torres F. J., Pérez C. M., Vergara J. L., Joshipura K. J. (2015). Lipid-lowering agents use and systemic and oral inflammation in overweight or obese adult Puerto Ricans: the San Juan Overweight Adults Longitudinal Study (SOALS). *Journal of Clinical Periodontology*.

[31] Cuschieri S. (2019). The STROBE guidelines. *Saudi Journal of Anaesthesia*.

[32] von Elm E., Altman D. G., Egger M. (2014). The strengthening the reporting of observational studies in epidemiology (STROBE) Statement: guidelines for reporting observational studies. *International Journal of Surgery*.

[33] Joshipura K., Muñoz-Torres F., Fernández-Santiago J., Patel R. P., Lopez-Candales A. (2020). Over-the-counter mouthwash use, nitric oxide and hypertension risk. *Blood Pressure*.

[34] Joshipura K. J., Muñoz-Torres F. J., Morou-Bermudez E., Patel R. P. (2017). Over-the-counter mouthwash use and risk of pre-diabetes/diabetes. *Nitric Oxide*.

[35] Sanz M., Marco Del Castillo A., Jepsen S. (2020). Periodontitis and cardiovascular diseases: consensus report. *Journal of Clinical Periodontology*.

[36] Caton J. G., Armitage G., Berglundh T. (2018). A new classification scheme for periodontal and peri-implant diseases and conditions—introduction and key changes from the 1999 classification. *Journal of Periodontology*.

[37] van der Velden U. (1984). Effect of age on the periodontium. *Journal of Clinical Periodontology*.

[38] Rivera R., Andriankaja O. M., Perez C. M., Joshipura K. (2016). Relationship between periodontal disease and asthma among overweight/obese adults. *Journal of Clinical Periodontology*.

